# CircRNAs: A promising target for intervention regarding glycolysis in gastric cancer

**DOI:** 10.1016/j.heliyon.2024.e34658

**Published:** 2024-07-15

**Authors:** Qian Dai, Yulin Liu, Fanghui Ding, Rong Guo, Gang Cheng, Hua Wang

**Affiliations:** a The First Hospital of Lanzhou University, Lanzhou, China, 730000; b The Second Clinical Medical College, Lanzhou University, Lanzhou, China, 730000

**Keywords:** Gastric cancer, Glycolysis, Warburg effect, CircRNA, miRNA

## Abstract

Gastric cancer is characterized by a high incidence and mortality rate, with therapeutic efficacy currently constrained by substantial limitations. Aerobic glycolysis in cancer constitutes a pivotal aspect of the reprogramming of energy metabolism in tumor cells and profoundly influences the malignant progression of cancer. CircRNAs, serving as stable endogenous RNA, have been shown to regulate downstream targets by sponging miRNAs, which in turn are involved in the regulation of multiple malignant behaviors in a variety of cancers through the CircRNA-miRNA axis, suggesting that CircRNAs could be used as potential therapeutic targets for cancer. In recent years, it has been shown that some CircRNAs can be involved in the regulation of GC glycolysis, therefore, this paper summarizes the notable roles of some important CircRNAs in the regulation of GC glycolysis in recent years, which may be useful for our understanding of GC progression and the development of new therapeutic strategies.

## 1 Introduction

Gastric cancer (GC) is a globally important disease, and according to epidemiological data, gastric cancer holds the fifth position in terms of incidence and the fourth position in mortality rates among cancers worldwide. In recent years, the incidence and mortality rates of gastric cancer have demonstrated a consistent upward trend in Asian countries, particularly within Southeast Asia, encompassing nations such as China, Japan, and Korea [1,2]. GC is a slow, multistage pathologic process caused by a combination of factors, including *Helicobacter pylori* infection, alcohol consumption, obesity, age, excessive salt and nitrate intake, and type A blood [2]. In addition, genetic mutations, epigenetic alterations and aberrant molecular signaling pathways play an important role in the development of GC [3,4]. Due to the lack of specific symptoms, the diagnosis of GC is often at a late stage. Currently, the diagnosis of GC mainly relies on imaging examination, serum tumor marker examination, gastroscopy, and tissue biopsy. Among these methods, gastroscopy combined with mucosal biopsy is considered the gold standard for GC diagnosis. The primary treatment for early-stage GC is endoscopic resection, while surgery constitutes the principal intervention for progressive GC. Chemotherapy is directed towards patients with advanced GC and those requiring adjuvant chemotherapy post-surgery. Additionally, immunotherapy and targeted therapy have demonstrated efficacy in the management of GC [5]. To varying degrees, these treatments have extended life expectancy and enhanced the prognosis of patients. Although we have made great progress in diagnostic and therapeutic means, the high recurrence rate and poor 5-year survival rate of GC make the prognosis of GC patients not optimistic, so it is significant to study the mechanism of malignant progression of GC to prolong the life span of the patients and improve the prognosis.

The phenomenon involving the energy supply of tumor cells has now been defined as metabolic reprogramming in tumor cells and has been classified as the eighth hallmark of tumors. Glycolysis plays a pivotal role in metabolic reprogramming, influencing not only glucose metabolism but also exerting effects on lipid and amino acid metabolism, as well as impacting mitochondrial function, apoptosis, and cell cycle progression [6]. Tumor cells prefer to acquire as much energy as possible for their own rapid growth rather than oxidative phosphorylation (OXPHOS). Glycolysis in tumor cells is known as aerobic glycolysis, or the Warburg effect, and is manifested primarily by increased glucose uptake, enhanced glycolytic activity, and increased lactate production. Song et al. experimentally found a significant increase in the tricarboxylic acid (TCA) cycle components such as fumarate and α-ketoglutarate in GC tissues, suggesting that there may be a Warburg effect shift in the GC [7]. Chen et al. demonstrated experimentally that the lactate content of GC cells was significantly increased, whereas citrate and succinate components were downregulated, suggesting an up-regulation of glycolysis and a decrease in the tricarboxylic acid (TCA) cycle in GC cells [8]. All of the above findings support the prevalence of the Warburg effect in patients with GC.

CircRNA is a stable endogenous circular RNA, abundant in mammals, and has been demonstrated as a prominent transcriptional entity in various human cells. CircRNAs have various mechanisms of action, mainly sponging miRNAs, binding to RNA binding proteins (RBPs), encoding proteins, and regulating selective splicing. miRNA is a non-coding single-stranded RNA molecule of 22 nucleotides in length, encoded by endogenous genes, which participates in the regulation of post-regulation gene expression by binding to the non-coding region at the 3′ end of its mRNA and accelerating the degradation of the 3′ end of the mRNA [9]. Presently, a diverse array of circRNAs has been demonstrated to exhibit abnormal expression in GC cells and tissues. These circRNAs function through the mechanism of sponging miRNAs, subsequently influencing the regulation of various aspects of GC biology, including proliferation, migration, invasion, metabolism, and radiotherapy resistance [10].

Despite abundant research reports suggesting that circRNAs can serve as potential targets for the clinical diagnosis and therapy of GC, there is a notable scarcity of studies focusing on glycolysis, a crucial aspect in the malignant progression of tumors. Hence, it is imperative to investigate the association between circRNAs and the regulation of GC development, particularly from the perspective of glycolysis. In this paper, we provide a summary of the substantial roles played by certain key circRNAs in the regulation of GC glycolysis in recent y.

## 2 Aerobic glycolysis in GC

### 2.1 The Warburg effect in GC

**Fig. 1:**
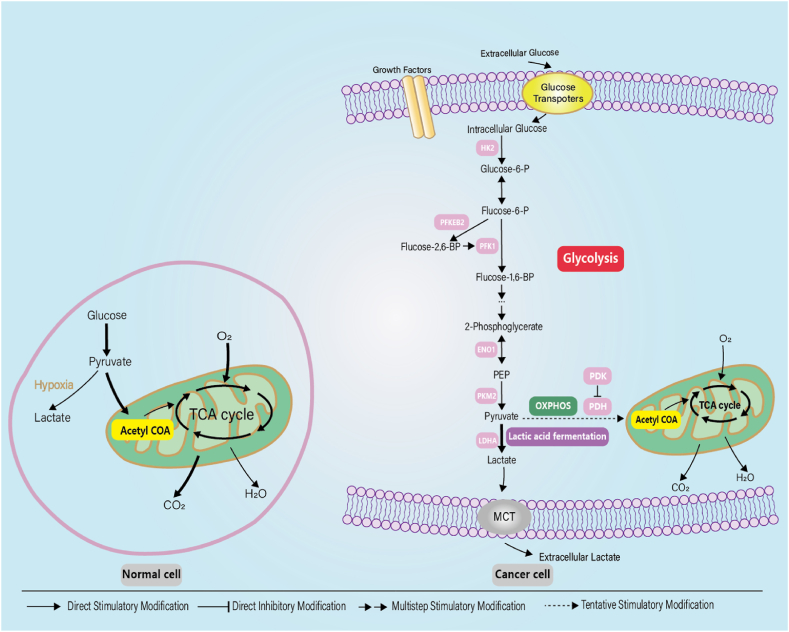
Schematic illustration of the differences between OXPHOS, anaerobic glycolysis and aerobic glycolysis (Warburg effect).

Normal cells utilize the tricarboxylic acid (TCA) cycle as the central hub for cellular glucose metabolism, where glucose is metabolized within the mitochondria, and energy is generated through oxidative phosphorylation (OXPHOS). Glycolysis, on the other hand, is typically employed as the primary metabolic pathway only under hypoxic conditions. However, in GC cells, the efficiency of ATP production through oxidative phosphorylation (OXPHOS) is notably low, while the rate of glycolysis exceeds that of normal cells by more than 200 times [11]. Even with sufficient oxygen, pyruvate from glycolysis does not enter the TCA cycle, but is catalyzed by LDH to produce lactate(Fig. 1). Under these changes, the efficiency of mitochondrial oxidative phosphorylation is reduced, and cancer cells have a significantly higher rate of glucose uptake, enhanced glycolytic activity, and increased lactate production. This phenomenon is commonly referred to as the Warburg effect in cancer cells, also known as aerobic glycolysis in cancer [12]. Consequently, assessing glucose consumption, lactate production, and ATP levels provides a valid indicator of cellular function and serves as a means to evaluate the glycolytic capacity of the cell.

Initially, Warburg himself held the belief that the capacity of tumor cells to sustain elevated rates of glycolysis originated from primary mitochondrial defects, specifically, irreversible respiratory damage. However, as research continued, it was discovered that when mitochondria functioned normally, oxidative phosphorylation (OXPHOS) in cancer cells proceeded as expected, while glycolysis remained upregulated. Eliminating the Warburg effect resulted in the glucose metabolism of cancer cells shifting towards OXPHOS. This implies flexibility in the transformation of mitochondrial metabolism, highlighting the existence of an energy metabolism continuum between mitochondrial OXPHOS and glycolysis [13,14].

The Warburg effect promotes the rapid uptake of glucose by cancer cells and its consumption through efficient glycolysis, which in turn generates large amounts of ATP to satisfy the energy needs of cancer cells and promotes cancer proliferation, migration, and invasion. On the other hand, a large amount of lactate produced by glycolysis can be transported to the extracellular area by MCTs, and the accumulation of lactate causes the TME to tend to a lower pH and to acidify, forming an extracellular acidic microenvironment. The extracellular acidic microenvironment activates transcription factors that upregulate PD-L1 on the surface of cancer cells, which in turn promotes immune escape of tumor cells through direct inhibition of T-cell activity and cytotoxicity of NK cells, among other effects [15]. Meanwhile, the diffusion of H+ secreted by cancer cells can enhance tumor invasiveness [12]. Secondly, the extracellular acidic microenvironment favors the upregulation of cytokines such as MMPs, which promotes cellular matrix remodeling and cell migration. In addition, lactic acid promotes tumor cell angiogenesis [16,17]. In turn, acidosis can upregulate the expression of key glycolysis factors through signaling pathways like the Wnt pathway, establishing a positive feedback loop that further enhances the Warburg effect. Furthermore, the Warburg effect contributes to a reduction in oxidative stress damage resulting from mitochondrial oxidative phosphorylation. In summary, aerobic glycolysis in tumor cells promotes proliferation, invasion, and metastasis to varying degrees through diverse pathways. In the case of GC cells, aerobic glycolysis induces tumorigenesis, fosters GC cell proliferation, and plays a role in mediating targeted therapy and resistance to chemotherapeutic agents. Therefore, the extent of aerobic glycolysis is closely intertwined with the rate of gastric cancer progression.

### 2.2 Mechanism of action of GC glycolysis and glycolysis-related factors

In tumor cells, the Warburg effect operates independently or synergistically through various mechanisms. The main mechanisms include: (a) Overexpression of the hypoxia-inducible factor HIF-1α; (b) Activation of oncogenes (e.g., c-Myc, mTORC1, Akt, and Ras); (c) Inactivation of tumor suppressors (e.g., p53 mutation); (d) Activation of signaling pathways such as receptor tyrosine kinase-PI3K-Akt-mTORC1 and Jak-Stat3; (e) Inactivation of the LKB1-AMPK signaling pathway; (f) Downregulation/loss of function of several miRNAs and SIRT3, SIRT6, etc.; and (g) Interaction with the hostile tumor microenvironment and cancer-associated stromal cell interactions [18]. The Warburg effect in GC is influenced not only by glycolysis but also by mitochondria, non-coding RNAs, and proteins that are not directly involved in the regulation of metabolism, etc. Alterations in the glycolytic pathway in GC result not only in glucose metabolism abnormalities but also in mitochondrial dysfunction, disturbances in the cell cycle, and irregularities in lipid and amino acid metabolism, among other factors [17]. Consequently, the glycolytic pathway of GC encompasses a variety of energy metabolism genes, enzymes, proteins, and transduction pathways. These key factors of glycolysis and signaling pathways collaborate to regulate the glucose metabolism of GC, subsequently impacting the proliferation, invasion, and metastasis of GC.

As a proto-oncogene, c-Myc stimulates the expression of glucose transporter protein 1 (GLUT1), as well as glycolysis-related enzymes such as HK2, LDHA, and PKM2, consequently promoting glycolysis [16,17]. The oncogene LKB1 can swiftly regulate the activity of G6P, a key enzyme in glycolysis, by activating AMPK, thereby enhancing glycolysis [19]. The inactivation of p53 can directly mediate the Warburg effect, while the upregulation of p53 inhibits GLUT1 and GLUT4 transcription and reduces the levels of F-2,6-BP, consequently inhibiting glycolysis through the induction of TP53 and TP53-induced regulator of apoptosis (TIGAR) [20]. GLUT1, HK2, PFK-1, PKM2, and LDHA serve as key rate-limiting enzymes in glycolysis, catalyzing the glycolytic pathway and representing direct targets of hypoxia-inducible factor 1-α (HIF-1α). HIF-1α exhibits high expression levels in GC and can directly enhance glycolysis by activating crucial glycolytic enzymes like GLUT1 and HK2 [12]. PDK1, identified as a direct target of Wnt, hinders pyruvate dehydrogenase (PDH) activity, thereby promoting the conversion of pyruvate to lactate and contributing to the regulation of the AKT/NF-κB pathway [21]. AMPK can detect changes in intracellular energy metabolism levels and regulate glucose metabolism [22]. The PI3K-Akt-mTOR pathway is activated in GC, promoting glycolysis and inhibiting autophagy [23]. The JNK signaling pathway stimulates glycolysis in GC cells and contributes to cancer cell proliferation [24]. The Wnt/β-catenin signaling pathway, one of the most crucial signaling pathways in tumor cell metabolism, can upregulate c-Myc, as well as the transcriptional levels of PDK1 and MCT1 [25]. Moreover, activation of the Wnt/β-catenin signaling pathway can accelerate glycolysis and enhance the malignant progression of GC through LRP5 [26].

## 3 CircRNA and GC

### 3.1 Biogenesis, characteristics and function of CircRNAs

CircRNA is an endogenous circular RNA that was first identified in the Sendai virus by electron microscopy in 1976 [27]. CircRNA is abundantly present in mammals and represents a significant transcriptional entity in various human cells [10]. Depending on their source, circRNAs can be categorized into four types: exonic circRNAs (EcRNAs), exon-intron circRNA (ElciRNAs), circular intronic RNAs (CiRNAs), and virus circRNAs [2,28]. There are two widely accepted models for CircRNA cyclization: lasso-driven cycling and intron pairing-driven cycling. The majority of these models result in the formation of EcRNAs or ElciRNAs, with the key distinction being whether introns between exons are preserved or not [29]. Conversely, CiRNAs are formed by reverse splicing inside the lasso precursor generated by exon skipping [30].

With the exception of intron-containing circRNAs, most circRNAs are transported from the nucleus to the cytoplasm after synthesis, facilitated by specific RNA helicases. As research has advanced, the biological characteristics of circRNAs have been elucidated. Due to its closed-loop structure, circRNA is less susceptible to nuclease degradation and exhibits greater stability than linear RNA. CircRNAs are widely distributed in eukaryotic cells, demonstrating diversity and high abundance. Furthermore, circRNAs are not only highly conserved across species but also exhibit tissue- and cell-specific and developmental stage-specific expression patterns [2].

Exploiting the biological characteristics of circRNA, numerous functions have been attributed to it. Given that circRNAs harbor multiple miRNA response elements (MREs), their most prominent function is to act as sponges for miRNAs. CircRNAs regulate miRNA function and the expression of related genes by competitively binding to miRNAs, thereby modulating the control of miRNAs on downstream targets [31]. Secondly, circRNAs can encode proteins. Despite lacking an efficient translation initiation structure and being categorized as non-coding RNAs, it has been demonstrated that circRNA can initiate translation when the eukaryotic ribosome inserts a ribosomal entry site (IRES) into circRNA. This suggests that circRNA is a potential template for protein translation [32]. Furthermore, circRNAs can bind to specific proteins, regulating their function, and the proteins interacting with circRNAs are known as RNA binding proteins (RBPs). It has also been observed that ElciRNAs and CiRNAs possess the ability to regulate the transcriptional activity of RNA polymerase II (Pol II). This, in turn, enables them to function as transcriptional regulators, modulating the expression of the parental genes [2].

### 3.2 CircRNA is involved in the development of GC

**Fig. 2:**
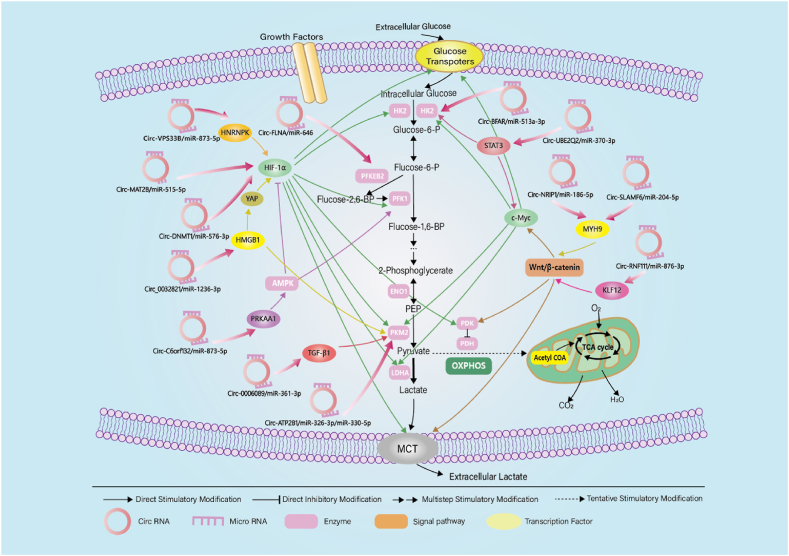
The CircRNAs mentioned in the article regulate the molecular mechanism of glycolysis in cancer cells.

Numerous studies have indicated that circRNAs exhibit abnormal expression in various tumor tissues. As investigations into the pathogenesis of GC progress, coupled with advancements in high-throughput sequencing and bioinformatics, an increasing number of circRNAs have been identified with abnormal expression in GC tissues, cells, blood and exosomes. Scientists have discovered that circRNAs can participate in the regulation of gene transcription and epigenetic modifications, thereby fulfilling diverse biological roles in tumors. These roles include promoting the proliferation, migration, and invasion of cancer cells, resisting apoptosis and inducing angiogenesis [33]. In general, abnormally upregulated circRNAs in the organisms of GC patients play a cancer-promoting role, whereas abnormally downregulated circRNAs inhibit cancer progression. For instance, CIRS-7 is significantly upregulated in GC tissues and promotes the migratory invasion of GC cells by acting as a sponge for miR-7, thereby antagonizing the miR-7-mediated PTEN/PI3K/AKT pathway [34]. Conversely, Circ-ZFR is expressed at low levels in GC tissues and cells, inhibiting GC tumor growth by sponging miR-130a/miR-107. This, in turn, increases the expression of PTEN and p53, ultimately promoting cancer cell apoptosis [35]. EMT is considered a crucial step in the early metastasis of cancer. The protein AXIN1-295aa, encoded by Circ-AXIN1, competes with the parental AXIN1 protein for binding to APC, subsequently releasing β-catenin. This activation of the Wnt/β-catenin signaling pathway promotes EMT in GC [36]. Angiogenesis is regarded as an initial hallmark of cancer metastasis. The absence of HIF-1α can induce decreased VEGF expression, impair vascular function. And Circ-RanGAP1, validated as a sponge for miR-877-3p, upregulates the expression of VEGFA. This stimulation of angiogenesis contributes to the promotion of GC metastasis [37]. In conclusion, circRNAs are widely involved in the malignant progression of GC. In recent years, numerous reports have suggested that circRNAs can additionally modulate glycolysis in GC cells by acting as miRNA sponges, thereby influencing GC development(Fig. 2).

## 4 CircRNA and glycolysis of GC

### 4.1 Circ-MAT2B

It has been demonstrated that Circ-MAT2B can promote glycolysis in liver cancer [38]. QRT-PCR showed that Circ-MAT2B was significantly upregulated in GC cells and tissues, while experiments confirmed its positive correlation with TNM staging in advanced GC. The xenograft tumor model confirms that the Circ-MAT2B gene inhibits tumor growth in vivo [39]. When Circ-MAT2B was knocked down, it was observed that the DNA synthesis rate of GC cells was significantly weakened, cell viability was significantly reduced, and glucose uptake and lactate production rates were drastically decreased, indicating that the knockdown of Circ-MAT2B gene inhibited the proliferation and glycolysis of GC cells [39]. Liu et al. found by experiment that miR-515-5p was lowly expressed in GC tissues, while down-regulation of the Circ-MAT2B gene could lead to a significant increase in the expression level of miR-515-5p. Upon silencing miR-515-5p in GC cells, the proliferation and glycolysis promoted by Circ-MAT2B were inhibited, indicating that Circ-MAT2B was able to sponge-bind and inhibit miR-515-5p in GC cells, resulting in accelerated proliferation and glycolysis in GC [39]. HIF-1α, a glycolysis-related gene, is significantly upregulated at the expression level in GC tissues, thereby promoting the glycolytic process in tumors [40]. The results of QRT-PCR showed that HIF-1α expression was significantly downregulated when miR-515-5p was overexpressed in GC cells, and this effect disappeared when miR-515-5p was knocked down, suggesting that HIF-1α is a downstream target of miR-515-5. In addition, it was also found that the knockdown of Circ-MAT2B significantly reduced the gene and protein levels of HIF-1α, while silencing of miR-515-5p rescued HIF-1α from the low expression state. Also, the overexpression of HIF-1α can directly promote the expression of Circ-MAT2B [39]. In summary, we can conclude that Circ-MAT2B promotes GC proliferation and glycolysis through the miR-515-5p/HIF-1α regulatory axis and that there is a positive feedback loop between Circ-MAT2B and HIF-1α.

### 4.2 Circ-DNMT1

It has been reported that Circ-DNMT is an autophagy modifier that can promote the malignant progression of breast cancer [41]. It was found that Circ-DNMT1 was highly expressed in GC tissues and cell lines and was closely associated with GC pathological staging. The knockdown of Circ-DNMT1 inhibited the proliferation and migration of GC cells and resulted in glucose uptake, lactate production, decreased ECAR, and increased OCR, indicating that Circ-DNMT1 promotes the proliferation, migration, invasion, and glycolysis of GC cells [42]. miR-576-3p has been shown to be lowly expressed in GC tissues, and bioinformatics analyses and dual luciferase reports indicate that Circ-DNMT1 inhibits miR-576-3p function through direct complementary binding. In vitro experiments showed that miR-576-3p overexpression inhibited GC proliferation, migration, invasion, and glycolysis metrics such as glucose uptake rate, while the overexpression of Circ-DNMT1 counteracted the inhibitory effect of miR-576-3p, suggesting that Circ-DNMT1 targeting miR-576-3p promotes GC progression and glycolysis. HIF-1α is a classical transcription factor that regulates glycolytic activity to sustain cancer survival and progression [43]. HIF-1α is highly expressed in GC cells and it has been reported that miR-576-3p can regulate HIF-1α expression in cancer [44]. Li et al. demonstrated experimentally that miR-576-3p directly inhibited HIF-1α expression in GC cells and antagonized the pro-proliferative and metabolic effects of HIF-1α in GC cells, and this antagonistic effect could not be reversed by the overexpression of Circ-DNMT1, while the silence of HIF-1α expression inhibited the promotion of Circ-DNMT1 on GC cell proliferation, migration and glycolysis [42]. In conclusion, we can suggest that Circ-DNMT1 promotes glycolysis and accelerates the malignant process of GC by targeting the miR-576-3p/HIF-1α axis.

### 4.3 Circ-SLAMF6

Circ-SLAMF6 is generated by reverse splicing of the first intron of SLAMF6. It has been shown to play an oncogenic role in bladder cancer by sponging miR-217 and regulating RUNX2 [45]. RT-PCR indicated that the expression of Circ-SLAMF6 was significantly upregulated in GC tissues. On this basis, Fang et al. experimentally found that the knockdown of the Circ-SLAMF6 gene suppressed glucose consumption, lactate production and HK2 protein expression in hypoxia-induced GC cells, and the cell migration invasion ability was weakened, indicating that Circ-SLAMF6 promotes glycolysis and invasive migration in hypoxic GC cells [46]. miR-204-5p has been shown to play an oncogenic role in a variety of cancers. Chen et al. demonstrated that LINC01234 promotes GC cell growth by sponging miR-204-5p [47]. Bioinformatics analysis showed the existence of a complementary binding site between Circ-SLAMF6 and miR-204-5p. Dual luciferase reporter and RIP assays indicated that the expression of miR-204-5p in GC cells and tissues was obviously reduced and that Circ-SLAMF6 was negatively correlated with the expression of miR-204-5p. In addition, Fang et al. found that inhibition of glycolysis and inhibition of migration and invasion due to silencing of Circ-SLAMF6 could be reversed by miR-204-5p inhibitors, suggesting that Circ-SLAMF6 promotes GC migration, invasion and glycolysis by targeting miR-204-5p [46]. MYH9 (myosin heavy chain 9) is a subtype of NMHCIIA (non-muscle myosin heavy chain IIA), and MYH9 has been shown to be expressed at significantly elevated levels in GC cells and tissues. And acts as an effector of the ROCK pathway, which promotes invasion and metastasis of GC cells [48]. It has been shown that MYH9 can mediate the ubiquitination degradation of GSK3β by recruiting the ubiquitin ligase TRAF6 and ultimately activate the Wnt/β-catenin pathway [49]. And the Wnt/β-catenin pathway is an essential component of aerobic glycolysis in tumor cells. Cao et al. demonstrated that Circ-ATP2A2 promotes glycolysis in osteosarcoma cells by sponging miR-335-5p and up-regulating MYH9 expression [50]. Fang et al. experimentally found that MYH9 knockdown significantly reduced the ability to migrate and invade, as well as the levels of glucose consumption, lactate production, and HK2 protein expression in hypoxic GC cells, suggesting that MYH9 promotes glycolysis in GCs and accelerates the malignant progression of cancer. In addition, MYH9 was found to be a downstream target of miR-204-5p and the overexpression of miR-204-5p in GC cells could inhibit the expression of MYH9, while the knockdown of Circ-SLAMF6 could reduce the expression of MYH9 protein, indicating that the expression of MYH9 was positively correlated with Circ-SLAMF6 and negatively correlated with miR-204-5p. In summary, we can conclude that Circ-SLAMF6 promotes GC proliferation, migration and glycolysis by regulating the miR-204-5p/MYH9 axis, but how exactly MYH9 regulates glycolysis levels still needs to be further explored.

### 4.4 Circ-NRIP1

According to reports, the expression of Circ-NRIP1 is upregulated in tissues and cells of GC patients, and Circ-NRIP1 can promote glucose consumption by sponging on miR-149-5p which targets AKT1 [51]. miR-186-5p has been shown to be downregulated in expression in a variety of cancers and acts as a tumor suppressor to regulate the biological functions of GCs in a low-expression manner. For example, the Circ-PDSS1/miR-186-5p/NEK2 axis promotes the gastric cancer cell cycle but inhibits apoptosis in vitro [52]. QRT-PCR showed that the expression of Circ-NRIP1 was upregulated and miR-186-5p was downregulated in the tissues and cells of patients with GC, and a negative correlation between the two was experimentally demonstrated in GC tissues [53]. Liu et al. observed that the expression of miR-186-5p was upregulated in GC cells when Circ-NRIP1 was experimentally knocked down. proliferation and migration of GC cells were inhibited, and the apoptotic process was accelerated. At the same time, glucose consumption, lactate consumption and ATP/ADP ratio were decreased in GC cells, and the expression of glycolysis rate-limiting enzymes such as HK2 and PKM2 was downregulated. And when only miR-186-5p was knocked down, all the above phenomena were reversed. Meanwhile, Western blotting showed that with the upregulation of Circ-NRIP1, HK2 and PKM2 protein levels were subsequently increased [53]. These all suggest that Circ-NRIP1 promotes GC cell proliferation, migration, and glycolysis and impedes apoptosis by directly sponging miR-186-5p. Dual luciferase report showing that MYH9 is a direct target of miR-186-5p in GC cells. When the expression of miR-186-5p was inhibited, the expression of MYH9 was upregulated, and the originally inhibited proliferation, migration and glycolysis processes of GC cells were re-accelerated, indicating that the relative expression of MYH9 was negatively correlated with the expression of miR-186-5p. And MYH9 was able to reverse the oncogenic effect of miR-186-5p [53]. It was also demonstrated that the overexpression of Circ-NRIP1 increased the protein level of MYH9 in GC cells [53]. All this evidence suggests that Circ-NRIP1 acts as an upstream regulator of the miR-186-5p/MYH9 axis, thereby promoting GC proliferation, migration and glycolysis.

### 4.5 Circ-VPS33B

The expression of Circ-VPS33B is upregulated in infiltrating GC tissues and cells [54]. And the high expression of Circ-VPS33B was associated with tumor size, TNM stage and lymphatic metastasis in GC patients. Through experiments, Liu et al. found that the knockdown of the Circ-VPS33B gene could lead to inhibition of migration and invasion, upregulation of E-calmodulin expression, reduction of ECAR, increase of OCR, and reduction of glucose uptake rate and lactate production rate in GC cells. This suggests that inhibition of Circ-VPS33B suppresses GC cell growth, proliferation, migration, invasion, EMT and Warburg effect [55]. The expression of miR-873-5p was downregulated in gastric cancer tissues. The experimental analysis suggested that miR-873-5p and Circ-VPS33B were negatively correlated in GC tissues, and the expression of miR-873-5p was upregulated in GC cells when Circ-VPS33B was silenced, whereas miR-873-5p did not have any effect on the expression of Circ-VPS33B. However, miR-873-5p knockdown reversed the inhibitory effects of Circ-VPS33B knockdown on GC cell proliferation and invasion, and the inhibitory effects on the rate of glucose uptake and the rate of lactate production in GC cells were also reversed [55]. This suggests that in GC cells, Circ-VPS33B regulates malignant processes and glycolysis by sponging miR-873-5p. HNRNPK is a kind of RNA-binding protein that has been shown to act as an oncogene involved in the regulation of a variety of biological processes and disease pathogenesis. Inhibition of HNRNPK increases post-irradiation DNA damage, thereby decreasing the survival of tumor cells [56]. Through experiments, Chen et al. found that HNRNPK can increase the expression of HIF-1α, which in turn promotes the expression of downstream glycolytic enzymes thereby promoting cellular glycolysis [57]. In GC tissues, the expression of HNRNPK protein was significantly upregulated, and experiments showed that when HNRNPK was overexpressed, the ECAR of GC cells was elevated, the OCR was decreased, the glucose uptake rate and lactate production rate of GC cells were elevated, and the glycolysis process was accelerated, and the inhibitory effects of miR-873-5p on the proliferation, invasion, EMT process, and Warburg effect of GC cells were reversed. These suggest that miR-873-5p regulates the degree of malignancy and Warburg effect in infiltrating GC cells by targeting HNRNPK [55]. In addition, Liu et al. found that HNRNPK mRNA expression in infiltrating GC tissues was negatively correlated with miR-873-5p but positively correlated with CircVPS33B, while miR-873-5p inhibitor reversed the down-regulation of HNRNPK in GC cells with knockdown of Circ-VPS33B. Altogether, we can conclude that Circ-VPS33B promotes tumor growth and the Warburg effect through the miR-873-5p/HNRNPK axis(Table 1).

**Table 1:** Changes in circRNAs involved in glycolysis in gastric cancer and other cancers.

CircRNA	Alteration	Target miRNA	miRNA target gene/portein	glycolysis	Other functions in GC	Cancer and/or other disorders
Circ-MAT2B	Up	miR-515-5p	HIF-1α	stimulative	Proliferation(+) Migration(+) Invasion(+)	Liver cancer
Circ-DNMT1	Up	miR-576-3p	HIF-1α	stimulative	Proliferation(+) Migration(+) Invasion(+)	Breast cancer
Circ-SLAMF6	Up	miR-204-5p	MYH9	stimulative	Proliferation(+) Migration(+) Invasion(+)	Bladder cancer
Circ-NRIP1	Up	miR-186-5p	MYH9	stimulative	Proliferation(+) Migration(+) Invasion(+) Apoptosis(−)	Ovarian cancer Cervical cancer Colorectal cancer ESCC
Circ-VPS33B	Up	miR-873-5p	HNRNPK	stimulative	Proliferation(+) Migration(+) Invasion(+) EMT(+)	
Circ-BFAR	Up	miR-513a-3p	HK2	stimulative	Proliferation(+)	PDAC LSCC GBM
Circ-FLNA	Up	miR-646	PFKFB2	stimulative	Proliferation(+) Migration(+) Apoptosis(−)	PTC LSCC
Circ-UBE2Q2	Up	miR-370-3p	STAT3	stimulative	Proliferation(+) Migration(+) Invasion(+) EMT(+)	
Circ-RNF111	Up	miR-876-3p	KLF12	stimulative	Proliferation(+) Migration(+) Invasion(+) Apoptosis(−)	Colorectal cancer Breast cancer SACC
Circ-0006089	Up	miR-361-3p	TGF-β1	stimulative	Proliferation(+) Migration(+) Angiogenesis(+) Apoptosis(−)	
Circ-ATP2B1	Up	miR-326-3p/miR-330-5p	PKM2	stimulative	Proliferation(+)	ccRCC
Circ-C6orf132	Up	miR-873-5p	PRKAA1	stimulative	Proliferation(+) Migration(+) Invasion(+)	
Circ-0032821	Up	miR-1236-3p	HMGB1	stimulative	Proliferation(+) Migration(+) Invasion(+) EMT(+)	Colorectal cancer

(+ means promoting, −means inhibiting).

### 4.6 Circ-BFAR

Circ-BFAR has been shown to promote pancreatic cancer by targeting miR-34b-5p/Met/Akt [58]. QRT-PCR showed that the expression of Circ-BFAR was significantly higher in GC cells and tissues than in normal tissues. CCK-8 assay and EDU assay showed that the knockdown of the Circ-BFAR gene significantly inhibited the proliferation of GC cells. When Circ-BFAR was knocked down, ECAR was downregulated and OCR was upregulated in GC cells, and metabolic reprogramming indicated that glycolysis was inhibited and extracellular lactate secretion was significantly reduced, suggesting that silence of Circ-BFAR inhibited glycolysis in GC cells [59]. Wang et al. demonstrated by experiments that Circ-BFAR could directly sponge miR-513a-3p in GC, inhibit miR-513a-3p expression and thus promote cell proliferation [59]. It has been reported that miR-513a-3p can directly target the 30UTR region of HK2 mRNA. miR-513a-3p negatively regulates HK2 in colorectal cancer, which in turn regulates glycolysis in CRC cells [60]. It was demonstrated by cell transfection experiments that Circ-BFAR abrogated the inhibitory effect of miR-513a-3p on HK2, suggesting that the expression of HK2 is regulated by the Circ-BFAR/miR-513a-3p axis [59]. Therefore, we can conclude that Circ-BFAR regulates glycolysis and growth and proliferation of GC cells by inhibiting the miR-513a-3p/HK2 axis.

### 4.7 Circ-FLNA

Through experiments, Qu et al. found that Circ-FLNA was upregulated in GC tissues and cells and was significantly associated with poor prognosis in GC patients. The silence of Circ-FLNA inhibited GC cell proliferation and metastasis, and promoted apoptosis [61]. The knockdown of Circ-FLNA inhibited lactate production, glucose consumption and ATP levels in GC cells, suggesting that Circ-FLNA promotes glycolysis in GC cells [61]. miR-646 is recognized as an anticancer factor that can be regulated by many pro-oncogenic cyclic RNAs. For example, Circ_0000267 can sponge miR-646 to promote the proliferation and metastasis of liver cancer [62]. In GC tissues and cells, the expression level of miR-646 was downregulated. Correlation analysis showed that Circ-FLNA was negatively correlated with miR-646. The overexpression of Circ-FLNA could significantly inhibit the expression of miR-646, suggesting that Circ-FLNA could directly interact with it by sponging miR-646 in GC. Meanwhile, miR-646 inhibitor was experimentally found to reverse the proliferation and invasion of GC cells inhibited by the silence of Circ-FLNA and promote apoptosis of GC cells. In addition, the miR-646 inhibitor also reversed the inhibitory effects of the silence of Circ-FLNA on lactate production, glucose consumption, ATP level and glucose uptake in GC cells [61]. The above results suggested that Circ-FLNA regulates the proliferation, metastasis, apoptosis and glycolysis of GC cells by acting as a sponge for miR-646. PFKFB2 is a bifunctional isomerase and an important regulatory molecule in glycolysis. The protein level of PFKFB2 was significantly enhanced in GC tissues and cells, and correlation analysis showed that the expression of PFKFB2 in GC was negatively correlated with the expression of miR-646 and positively correlated with the expression of Circ-FLNA. Western blotting showed that overexpression of miR-646 inhibited PFKFB2 expression, and miR-646 inhibitor also reversed the inhibitory effect of Circ-FLNA silencing on PFKFB2. In contrast, the overexpression of PFKFB2 also reversed the inhibitory effects of miR-646 on GC cell proliferation and invasion as well as on lactate production, glucose consumption, ATP levels, and glucose uptake [61]. In summary, Circ-FLNA regulates the miR-646/PFKFB2 signaling axis, which in turn regulates the process of GC growth and glycolysis.

### 4.8 Circ-UBE2Q2

Circ-UBE2Q2 was detected to be highly expressed in GC tissues by QRT-PCR, and Yang et al. found that Circ-UBE2Q2 was mainly localized in the cytoplasm of GC cells and that Circ-UBE2Q2 promoted the proliferation, invasion, migration, and EMT process of GC cells, and even the growth of gastric organoid model [63]. miR-370-3p was also localized in the cytoplasm of GC cells and its expression was significantly upregulated in GC tissues. The dual luciferase report showed a positive targeting relationship between Circ-UBE2Q2 and miR-370-3p. QRT-PCR showed that the inhibition of proliferation and metastasis induced by the knockdown of Circ-UBE2Q2 could be successfully reversed by the silence of miR-370-3p. Simultaneous knockdown of miR-370-3p and Circ-UBE2Q2 revealed that the growth of a GC-like organoid model blocked by the knockdown of Circ-UBE2Q2 was significantly reversed [63]. These all suggest that Circ-UBE2Q2 can act as a sponge for miR-370-3p to promote GC development.

STAT3 is considered an oncogene in the STAT protein family and is associated with cellular autophagy, cell cycle, glycolysis and metastasis. In GC, STAT3 targets the promoter of HK2, upregulates HK2 gene expression and promotes glycolysis [64]. It has been found that the activation of STAT3 can regulate the downstream target gene c-Myc and thus activate the STAT3/c-Myc signaling pathway, which interacts with the mTOR/PKM2 signaling pathway in GC cells to regulate the glycolysis and acidic microenvironment in tumor cells [65]. It was also found that the activation of atypical STAT3 is a key signaling mediator of TLR4-induced glycolysis, macrophage metabolic reprogramming, and inflammation [66]. All this evidence suggests that the activation of STAT3 can promote glycolysis in tumor cells. Survival analysis and immunohistochemical experiments revealed that STAT3 was significantly upregulated in GC tissues and the expression level of STAT3 was significantly negatively correlated with miR-370-3p. Dual luciferase report confirmed that STAT3 was a downstream target of miR-370-3p. A positive correlation between Circ-UBE2Q2 and STAT3 was found by qRT-PCR, and the knockdown of Circ-UBE2Q2 inhibited STAT3 expression in GC cells, while the suppression of GC cell proliferation and metastasis caused by the silence of Circ-UBE2Q2 could be reversed by the overexpression of STAT3 [63]. GSEA analysis showed that STAT3 was significantly correlated with glycolysis, and when the Circ-UBE2Q2 gene was knocked down, the efficiency of ECAR and glycolysis was decreased, and the expression of enzymes for glycolysis such as HK2 and PFK was downregulated, whereas the overexpression of STAT3 reversed this change [63]. The above evidence suggests that Circ-UBE2Q2 inhibits tumor autophagy and promotes GC proliferation, migration, and glycolysis processes through the miR-370-3p/STAT3 axis.

### 4.9 Circ-RNF111

Circ-RNF111 is highly expressed in GC cells and tissues, and tumor growth in vivo is inhibited when the Circ-RNF111 gene is knocked down [67]. Wu et al. found that the levels of glucose consumption, lactate production, ATP production, and HK-2 protein were reduced in GC cells when Circ-RNF111 was deficient, suggesting that glycolysis was inhibited in GC cells after the knockdown of this gene [67]. QRT-PCR showed that the level of miR-876-3p was reduced in GC cells, and the level of miR-876-3p in GC tissues was negatively correlated with the level of Circ-RNF111. Circ-RNF111 and miR-876-3p were found to share a binding site, and dual luciferase reporter and RIP assays indicated an interaction between Circ-RNF111 and miR-876-3p. When Circ-RNF111 was silenced, miR-876-3p expression was upregulated, GC cell growth, metastasis and glycolysis were inhibited, the cell cycle was blocked and apoptosis was accelerated. While the opposite result was shown when Circ-RNF111 was overexpressed [67]. The above evidence suggests that Circ-RNF111 can inhibit miR-876-3p expression by sponging miR-876-3p, which in turn promotes the malignant process of GC cells.

The family of KLFs has been reported to be involved in the development of a variety of cancers, of which KLF8 has been shown to induce glycolysis and angiogenesis in GC by acting on GLUT4 [68]. In addition, it was found that KLF12, as a downstream target of miR-137, could participate in tumor cell glycolysis by activating the Wnt/β-catenin pathway through the miR-137/KLF12 axis [69]. Bioinformatics analysis revealed that KLF12 is a target gene of miR-876-3p. KLF12 was highly expressed in GC cells, and the level of KLF12 mRNA was negatively correlated with the level of miR-876-3p in GC tissues. The overexpression of miR-876-3p decreased the KLF12 protein level, and the levels of glucose uptake, lactic acid production, ATP production in GC cells and HK2 protein levels decreased. In contrast, KLF12 up-regulation showed the opposite effect, indicating that the overexpression of miR-876-3p inhibited GC cell growth, metastasis and glycolysis, promoting apoptosis and cell cycle arrest by targeting KLF12 [67]. Wu et al. found that the silence of Circ-RNF111 resulted in downregulation of KLF12 protein levels, which was reversed by the silence of miR-876-3p, suggesting that Circ-RNF111 positively regulated KLF12 expression by sponging miR-876-3p [67]. In summary, we can conclude that Circ-RNF111 regulates the growth, proliferation and glycolysis processes of GC by modulating the miR-876-3p/KLF12 axis, but how exactly KLF12 regulates the glycolysis level remains to be further explored.

### 4.10 Circ-0006089

Circ-0006089 has been shown to be significantly upregulated in GC. Zhou et al. predicted the downstream miRNA targets of Circ-0006089 by Bioinformatics analysis. QRT-PCR revealed that the expression of miR-361-3p was downregulated in GC tissues and negatively correlated with that of Circ-0006089. When Circ-0006089 was knocked down, the expression of miR-361-3p was significantly upregulated. Functional analysis showed that the knockdown of Circ-0006089 inhibited proliferation, migration, glucose consumption, lactate production, ATP levels and HK2 protein expression in GC cells. And miR-361-3p inhibitor reversed these effects, proving that miR-361-3p inhibited the malignant progression of GCs [70]. In summary, we can suggest that Circ-0006089 promotes proliferation, migration and glycolysis and other malignant progression of GC by sponging miR-361-3p. TGF-β1 belongs to the TGF-β subfamily and has been shown to be widely involved in a variety of pathological processes in cancer. For example, TGF-β1 can act as a protein kinase, induce PKM2 and then activate the PKM2/STAT1-PD-L1 axis, which promotes the process of tumor glycolysis, remodels the immune microenvironment, and promotes tumor proliferation, migration and invasion [71]. The expression level of TGF-β1 in GC tissues was confirmed to be elevated and negatively correlated with the expression of miR-361-3p. Western blotting showed that miR-361-3p mimics could inhibit the expression of TGF-β1 proteins, indicating that TGF-β1 is a downstream target of miR-361-3p [70]. It was found that the inhibitory effect of miR-361-3p on GC growth, migration, and glycolysis could be reversed by the overexpression of TGF-β1, suggesting that miR-361-3p inhibited the growth, metastasis, and glycolysis of GC cells by targeting TGF-β1 [70]. In GC tissues, the expression of TGF-β1 mRNA was positively correlated with that of Circ-0006089. The overexpression of Circ-0006089 significantly enhanced the gene and protein expression of TGF-β1 in GC cells, and this effect could be reversed by miR-361-3p mimics, demonstrating that Circ-0006089 positively regulates TGF-β1 by sponging miR-361-3p [70]. In summary, we can conclude that Circ-0006089 regulates GC growth, proliferation, and glycolysis by modulating the miR-361-3p/TGF-β1 axis.

### 4.11 Circ-ATP2B1

QRT-PCR confirmed that Circ-ATP2B1 was highly expressed in GC cells and tissues, and it was found that the expression of GLUT1, GLUT3, LDHA, and PKM2 in GC tended to increase with the increase of pathological grading of GC, which was consistent with the trend of the expression of Circ-ATP2B1 [72]. When Circ-ATP2B1 was overexpressed, the viability, glucose and lactate production, and ATP/ADP ratio of GC cells were increased, while the above phenomena were suppressed when the gene was knocked down [72], suggesting that Circ-ATP2B1 promotes the proliferation and glycolysis of GC cells. The expression of miR-326-3p/miR-330-5p was downregulated in GC cells, and GO analysis revealed that the miR-326 gene cluster (miR-326-3p/miR-330-5p) is a downstream target of Circ-ATP2B1, and the cluster is functionally focused on cell growth and metabolism [73]. The expression of miR-326-3p/miR-330-5p was found to be significantly downregulated in Circ-ATP2B1 overexpressing GC cells. When Circ-ATP2B1 was knocked down, miR-326-3p/miR-330-5p was overexpressed, and the proliferation, migration, and glycolysis of GC cells were inhibited, indicating that Circ-ATP2B1 promotes glycolysis by targeting miR-326-3p/miR-330-5p [72]. PKM2 acts as a rate-limiting enzyme of glycolysis, accelerating glucose uptake by GC cells and catalyzing glycolysis. Immunohistochemistry showed that PKM2 was highly expressed in GC and positively correlated with the pathological grade of GC, suggesting that PKM2 promoted the malignant progression of GC. It was found that when PKM2 was silenced, the proliferation, glucose uptake, lactate production, and ATP of GC cells were inhibited. PKM2 has been shown to be a potential target gene for miR-326-3p/miR-330-5p [74]. Zhao et al. found that after silencing Circ-ATP2B1, the expression of PKM2 was significantly reduced and glycolysis of GC was slowed down. However, after miR-326-3p/miR-330-5p silencing, the expression of PKM2 was increased and the glycolysis of GC was accelerated [72]. The above evidence suggests that Circ-ATP2B1 regulates glycolysis in GC cells by targeting the miR-326-3p/miR-330-5p/PKM2 axis.

### 4.12 Circ-C6orf132

QRT-PCR showed that the expression of Circ-C6orf132 was upregulated in GC cells under hypoxic conditions. It was shown that the proliferation, glucose production, lactate production and ATP levels of hypoxia-induced GC cells were significantly increased when the expression of Circ-C6orf132 was upregulated. When silencing Circ-C6orf132, the above changes tended to be suppressed, indicating that Circ-C6orf132 significantly promoted the proliferation, migration, invasion and glycolysis process of GC cells [75]. In addition, Chen et al. found that miR-873-5p was lowly expressed in GC cells and that the expression of Circ-C6orf132 was negatively correlated with miR-873-5p. The knockdown of Circ-C6orf132 promoted the expression of miR-873-5p in GC cells, suggesting that miR-873-5p is a downstream target of Circ-C6orf132. In contrast, the proliferation, migration and glycolysis promoted in GC cells due to the high expression of Circ-C6orf132 could be reversed by the addition of miR-873-5p inhibitor, suggesting that Circ-C6orf132 achieves the promotion of GC growth and metabolism by sponging miR-873-5p [75]. AMPK (5′-AMPK-activated protein kinase) can mediate glycolysis in GC cells by phosphorylating the activity of key enzymes of glycolysis. PRKAA1 (protein kinase AMP-activated α1 catalytic subunit), a subunit of AMPK, has been shown to be overexpressed in GC tissues and promote energy metabolism and tumor progression in GC [76]. Dual luciferase reporter analysis showed that miR-873-5p could bind to PRKAA1. Linear analysis of GC tissues showed that miR-873-5p was negatively correlated with PRKAA1, while Circ-C6orf132 was positively correlated with PRKAA1. Western blotting showed that the overexpression of miR-873-5p directly downregulated PRKAA1 [75]. The above evidence suggests that with PRKAA1 is a direct downstream target of miR-873-5p. Furthermore, it was demonstrated that the inhibition of GC cell proliferation and migration and the decrease of gluconeogenesis, lactate production, ATP levels, and GULT1/HK2 levels caused by the high miR-873-5p expression induced by knockdown of Circ-C6orf132 could be reversed by the overexpression of PRKAA1 [75]. These suggest that Circ-C6orf132 inhibits GC proliferation, migration and glycolysis under hypoxic conditions by targeting the miR-873-5p/PRKAA1 axis.

### 4.13 Circ-0032821

Circ-0032821 has been shown to promote a malignant phenotype in GC cells by activating the MEK1/ERK1/2 signaling pathway [77]. QRT-PCR showed that the expression of Circ-0032821 was significantly upregulated in GC tissues. And in vivo, experiments also confirmed the oncogenicity of Circ-0032821. When Circ-0032821 was knocked down, the proliferation, migration and invasion ability of GC cells were significantly inhibited, the glucose uptake rate and lactate content, etc. were significantly reduced, and the ECAR of GC tissues was decreased [78], suggesting that Circ-0032821 could promote the proliferation, migration, invasion and glycolysis process of GC cells. The oncogenic effects of miR-1236-3p have been demonstrated in a variety of cancers. For example, miR-1236-3p inhibits GC invasion and migration through interaction with mTA2 [79]. In GC tissues and cells, miR-1236-3p was lowly expressed, and experiments showed that the overexpression of miR-1236-3p significantly inhibited the malignant process of GC. Chen et al. identified miR-1236-3p as a downstream target of Circ-0032821. Experiments revealed that transfection of circ_0032821 significantly reduced miR-1236-3p levels in GC cells [78]. When Circ-0032821 was knocked down, the proliferation migration and invasion ability of GC cells were significantly inhibited, the glucose uptake rate and lactate content, etc. were significantly reduced, and the ECAR of GC tissues was decreased [78], suggesting that Circ-0032821 could promote the proliferation, migration, invasion, and glycolysis of GC cells. And when miR-1236-3p was knocked down, the above phenomena could be reversed [78]. Therefore, we can conclude that Circ-0032821 negatively regulates the expression of miR-1236-3p by sponging miR-1236-3p, thereby promoting GC growth and glycolysis. High mobility group protein 1 (HMGB1) is a kind of non-histone chromosomal protein. And it has been found that HMGB1 inhibits cellular aerobic respiration by denaturing the tetrameric form of PKM2 without affecting other glycolytic enzymes thereby inhibiting cellular aerobic respiration and forcing the cell to undergo dimerization-mediated glycolysis of PKM2 [80]. Meanwhile, HMGB1 has been shown to play an important role in the development of GC. For example, HMGB1 can regulate the YAP-mediated HIF-1α pathway and activate the transcription of glycolysis-related genes, which in turn induces glycolysis and promotes the malignant progression of tumor [81]. Chen et al. found that the expression of HMGB1 was significantly upregulated in GC tissues and cells and that HMGB1 was a direct target of miR-1236-3p [78]. In addition, the mRNA level of HMGB1 was found to be negatively correlated with miR-1236-3p level and positively correlated with Circ-0032821. The overexpression of MiR-1236-3p attenuated the malignant behavior of GC cells by targeting HMGB1, while the overexpression of HMGB1 reversed the inhibitory effects of miR-1236-3p on GC proliferation, migration and glycolysis [78]. The knockdown of Circ_0032821 significantly downregulated the expression of HMGB1 in GC cells, and this downregulation could be attenuated when miR-1236-3p was inhibited [78]. In conclusion, we can summarize that Circ_0032821 promotes the proliferation, migration, invasion and glycolysis of GC cells by targeting the expression of miR-1236-3p/HMGB1 axis.

## 5 Conclusion and perspective

As a cancer with a high incidence and mortality rate, GC has witnessed significant progress in diagnostic and therapeutic approaches. However, the invasive nature of diagnostic methods, patient discomfort during the diagnostic process, and delays in diagnosis highlight the need for novel diagnostic approaches in clinical settings. Simultaneously, the side effects of GC chemotherapy and the resistance of GC to chemotherapeutic drugs underscore the necessity to explore safer, more reliable, and more efficient treatments in the future. Aerobic glycolysis in cancer cells, serving as a major mode of efficient energy acquisition by tumors, has emerged as a core aspect of metabolic reprogramming in cancer. It constitutes a key component of the malignant phenotype of cancer and is considered one of the hallmarks of cancer [82]. It has been discovered that aerobic glycolysis in cancer cells results from the independent or synergistic actions of multiple genes, transcription factors, proteins, and signaling pathways. Moreover, it is regulated by a variety of factors [18]. CircRNAs, as abundantly present transcriptional substances in human cells, have demonstrated their involvement in the development of various cancers through diverse channels and to varying degrees. CircRNAs have been identified as participants in aerobic glycolysis in tumors by engaging in mechanisms such as sponging miRNAs, translating proteins and binding to RBPs among others [15]. In this review, we focus on summarizing certain circRNAs that influence glycolysis in GC cells through the circRNA-miRNA regulatory network, with the anticipation of providing new diagnostic and therapeutic targets for GC.

On one hand, circRNAs exhibit abundant expression in GC cells, tissues, and body fluids, making them highly detectable. Additionally, circRNA, being a circular RNA, demonstrates greater stability compared to typical linear RNA and is less prone to degradation. Consequently, circRNA is well-suited as an independent marker for diagnosis and prognosis. On the other hand, aberrantly expressed circRNAs in tumor cells can precisely interfere with tumor cells with minimal impact on normal cells in the body. Consequently, circRNAs can also serve as precision therapeutic targets for tumors. With the continuous discovery of more circRNAs associated with the regulation of the Warburg effect in tumors, it is imperative to persist in our research efforts to facilitate the translation of these circRNAs into clinically viable diagnostic markers and therapeutic targets.

However, there are still numerous obstacles regarding the translational clinical potential of circRNAs. Concerning circRNA itself, we still lack a clear enough understanding of the mechanisms of circRNA biogenesis, translocation, dysregulation of expression, and how circRNAs are degraded. Regarding circRNA therapy, many issues also require further exploration. Firstly, circRNA is typically achieved by expressing plasmids or synthesizing and purifying circRNA to attain circRNA overexpression, and circRNA knockdown is achieved through RNA interference-based strategies. In recent years, advancements have been made in the regulation of CircRNA through various methods such as the Cre-Lox system, CRISPR/Cas9 system, CRISPR/Cas13 system, etc. Additionally, there is growing interest in leveraging exosomes, nanoparticles, liposomes, lentiviruses, and adenoviruses as potential delivery systems for interfering with the expression of circRNAs [83]. However, it is crucial to note that different delivery systems come with varying degrees of limitations. Thus, the selection of an appropriate delivery system is paramount for enhancing the efficacy of circRNA therapy. Secondly, we must take into account the repercussions of targeted interventions on the corresponding genes and linear transcripts of the circRNAs. It is essential to assess whether these interventions will have an impact on the organism and potentially influence the therapeutic outcome. Thirdly, apart from the established mechanisms governing glycolysis regulation, circRNAs may also exert control over glycolysis through other transcription factors and signaling pathways that operate within a complex network. The extent to which interference with circRNAs may be counteracted by alternative mechanisms remains unclear. Fourthly, while the majority of CircRNAs exhibit tissue- or cell-specific expression patterns, certain CircRNAs may be linked to multiple diseases. For instance, Circ-NRIP1 has been implicated in various conditions including gastric cancer, ovarian cancer, osteosarcoma, esophageal squamous cancer etc. [53,[84], [85], [86]]. Therefore, interfering with circRNAs may introduce potential off-target effects, potentially influencing therapeutic efficacy.

In conclusion, the clinical application of circRNAs in treatment is still in the early stages. Despite a limited number of circRNAs associated with glycolysis in GC being thoroughly investigated, and specific mechanisms requiring further exploration, there is a growing suggestion that these circRNAs could potentially serve as therapeutic targets for GC glycolysis. As we continue to unravel the intricate details of circRNA involvement in GC glycolysis, there remains optimism regarding their potential as valuable therapeutic candidates.

## Fundings

This work was supported by the 2022 Hospital Internal Funded Program for the First Hospital of 10.13039/100012899Lanzhou University (ldyyyn2022-21) and the 2024 Gansu Province Education Science and Technology Innovation Project(2024B-015).

## Institutional review board statement

Not applicable.

## Informed consent statement

Not applicable.

## Data availability statement

Not applicable.

## CRediT authorship contribution statement

**Qian Dai:** Writing – original draft, Funding acquisition. **Yulin Liu:** Writing – original draft. **Fanghui Ding:** Writing – original draft. **Rong Guo:** Writing – review & editing. **Gang Cheng:** Writing – original draft. **Hua Wang:** Funding acquisition.

## Declaration of competing interest

The authors declare that they have no known competing financial interests or personal relationships that could have appeared to influence the work reported in this paper.
